# The Tell-Tale Heart: Population-Based Surveillance Reveals an Association of Rofecoxib and Celecoxib with Myocardial Infarction

**DOI:** 10.1371/journal.pone.0000840

**Published:** 2007-09-05

**Authors:** John S. Brownstein, Margarita Sordo, Isaac S. Kohane, Kenneth D. Mandl

**Affiliations:** 1 Children's Hospital Informatics Program at the Harvard-MIT Division of Health Sciences and Technology, Boston, Massachusetts, United States of America; 2 Division of Emergency Medicine, Children's Hospital Boston, Boston, Massachusetts, United States of America; 3 Department of Pediatrics, Harvard Medical School, Boston, Massachusetts, United States of America; 4 Decision Systems Group, Brigham and Women's Hospital, Boston, Massachusetts, United States of America; 5 Center for Biomedical Informatics, Harvard Medical School, Boston, Massachusetts, United States of America; 6 Department of Medicine, Brigham and Women's Hospital, Boston, Massachusetts, United States of America; York University, Canada

## Abstract

**Background:**

COX-2 selective inhibitors are associated with myocardial infarction (MI). We sought to determine whether population health monitoring would have revealed the effect of COX-2 inhibitors on population-level patterns of MI.

**Methodology/Principal Findings:**

We conducted a retrospective study of inpatients at two Boston hospitals, from January 1997 to March 2006. There was a population-level rise in the rate of MI that reached 52.0 MI-related hospitalizations per 100,000 (a two standard deviation exceedence) in January of 2000, eight months after the introduction of rofecoxib and one year after celecoxib. The exceedence vanished within one month of the withdrawal of rofecoxib. Trends in inpatient stay due to MI were tightly coupled to the rise and fall of prescriptions of COX-2 inhibitors, with an 18.5% increase in inpatient stays for MI when both rofecoxib and celecoxib were on the market (P<0.001). For every million prescriptions of rofecoxib and celecoxib, there was a 0.5% increase in MI (95%CI 0.1 to 0.9) explaining 50.3% of the deviance in yearly variation of MI-related hospitalizations. There was a negative association between mean age at MI and volume of prescriptions for celecoxib and rofecoxib (Spearman correlation, −0.67, P<0.05).

**Conclusions/Significance:**

The strong relationship between prescribing and outcome time series supports a population-level impact of COX-2 inhibitors on MI incidence. Further, mean age at MI appears to have been lowered by use of these medications. Use of a population monitoring approach as an adjunct to pharmacovigilence methods might have helped confirm the suspected association, providing earlier support for the market withdrawal of rofecoxib.

## Introduction

In the United States, almost a million people are hospitalized, and 200,000-300,000 die each year of myocardial infarction (MI).[1], [2] Rates of MI have increased over the last century and are attributable to lifestyle risk factors, including smoking, obesity, exercise and diet.[3], [4], [5] While non-cardiovascular target drugs such as fenfluramine, dexfenfluramine, terfanadine and cisapride have been shown to be risk factors for cardiovascular events [6], [7], [8], their effects are rare and are not considered as contributors to long term trends in morbidity and mortality.

Nonsteroidal anti-inflammatory drugs (NSAIDs) are increasingly implicated in cardiovascular morbidity [9] and Cyclooxygenase-2 (COX-2) selective inhibitors, a class of NSAIDs, have been, in particular, associated with increased risk of MI.[9], [10], [11], [12], [13], [14], [15], [16] Rofecoxib was withdrawn from the market after a randomized placebo-controlled trial revealed increased cardiovascular risk in patients with colorectal polyps.[17] Though similarly associated with cardiovascular risk [9], celecoxib is still prescribed widely. Many studies have supported an increased individual risk of MI, but the population-level impact of COX-2 selective inhibitor prescription has not been described or quantified despite their substantial market penetration and delayed market withdrawal.

In this study, we elucidate long-term temporal trends in rates of inpatient visits for MI to an integrated health system in Boston, Massachusetts and their correspondence with prescriptions for rofecoxib and celecoxib. We estimate the magnitude of this effect on macro-level trends in hospitalizations for MI.

## Methods

### Study Setting and Population

The patient population was from Partners Healthcare System, a large multi-specialty group practice that provides care to members of a New England–based health maintenance organization. This non-profit, integrated health system includes Brigham and Women's Hospital and Massachusetts General Hospital. The source of clinical data—patient demographic information, dates, medication, and diagnosis information, and discharge summaries—was the Research Patient Data Registry (RPDR), a centralized data warehouse. Institutional Review Board (IRB) approval was obtained.

The study outcome was serious coronary heart disease, defined as acute MI requiring hospitalization. Subjects were all patients hospitalized for MI assigned by primary or admitting diagnosis International Classification of Diseases, Ninth Revision (ICD-9) code 410, from January 1 1997 to March 31 2006. This diagnostic code has been validated by others and used as an indicator of acute MI. [10], [18], [19] In our own chart review, we found that 87% of patients assigned the code had strong confirmatory evidence of acute MI. To calculate incidence, we estimated the denominator by combining US Census population estimates for Massachusetts from 1997–2005 with market share estimates for the two study hospitals. Market share was calculated using data from the Massachusetts Division of Health Care Finance and Policy (1999–2004) which contains hospitalizations at all Massachusetts hospitals. Mean yearly market share was estimated at 12.4% (SD, 0.6%). To adjust for biases stemming from shifts in healthcare utilization patterns and changes in health coverage and market share, we also included total number of hospitalizations per unit time as a covariate in our analyses.

### Interrupted Time Series Analysis

Using a population monitoring framework, we tested for a change in the population rate of MI after introduction of rofecoxib and celecoxib using three time series analysis methods. The first was cumulative sum (CUSUM), regularly used in industrial process control. More recently it has been applied to the early detection of disease outbreaks [20] and to adverse drug events.[21], [22] The CUSUM, a type of control chart, detects sudden changes in the mean value of a quantity of interest and provides estimates of the timing and magnitude of change.[23] Specifically, the analysis calculates the cumulative sum of deviations from a set value in successive samples. An alarm threshold value is defined a priori taking into account an acceptable false alarm rate or average run length. Here, we used CUSUM to define whether and when a detectable rise in cardiovascular events could have been identified by simply monitoring the time series of monthly rate of inpatient stays for MI.

Second, we investigated the association of the market availability of celecoxib (January 1999–present), and rofecoxib (May 1999–September 2004) on the time series of the monthly rate of inpatient stays for MI using generalized linear modeling. We assumed a Poisson distribution for modeling counts of independent MI events. The model included binary indicator variables for rofecoxib and celecoxib (on or off the market), and used estimated population covered as the offset. Seasonal trends in MIs were adjusted for by including eleven months of the year as indicator variables. We included monthly number of total inpatient stays to adjust for potential secular trends in healthcare utilization. A linear trend term was included to account for long-term changes in MI-related hospitalizations. To account for overdispersion in the count data, extra-Poisson variability was modeled and incorporated into estimates of standard errors.[24] Parameter estimates were transformed to rate ratios.

Finally, we matched fluctuations in yearly incidence of hospitalizations due to MI to prescription data on rofecoxib and celecoxib. Total numbers of prescriptions dispensed were obtained from the freely available IMS Health annual report on Commonly Requested Therapeutic Class and Product Information.[25] We measured the impact of celecoxib and rofecoxib prescription rates by fitting Poisson regression models as described above. In this case, we modeled yearly counts of MIs, including total underlying population as an offset, and overall number of yearly inpatient stays as a model covariate. A linear trend term was included to account for long-term secular trends.[26] Prescription counts for celecoxib and rofecoxib were examined separately and in combination. In addition to incidence, we also examined the association between yearly changes in the mean patient age at time of MI with prescription volume for rofecoxib. All analyses were carried out using the SAS statistical software (version 9.0, SAS Institute Inc., Cary, NC).

### Anchor Diagnoses

To confirm that our results were not simply reflective of systematic bias related to referral patterns and secular trends in acuity mix at the two hospitals, we monitored yearly changes in three anchor diagnoses. We investigated whether yearly incidence of hospitalizations due to abdominal aortic aneurysm (ICD-9 codes 441.3–441.4), appendicitis (ICD-9 codes 441.3–441.4), and pneumonia and influenza (ICD-9 codes 480–487) were coincident with MIs by two-tailed Spearman rank correlation.

## Results

### Study Population

We identified 25,486 unique patients who had 30,603 MI-related hospitalizations during the study period. Monthly incidence of hospitalizations due to MI ranged from 33.3 per 100,000 inpatient stays in March of 2006 to 56.4 per 100,000 in December, 2002 (mean, 46.2; 95 percent confidence interval, 45.1 to 47.2). Incidence of hospitalizations due to MI ranged from 48.8 per 10,000 in 1997 and 2005 to 62.3 per 10,000 in 2001.

### Interrupted Time Series Analysis

**Figure 1:**
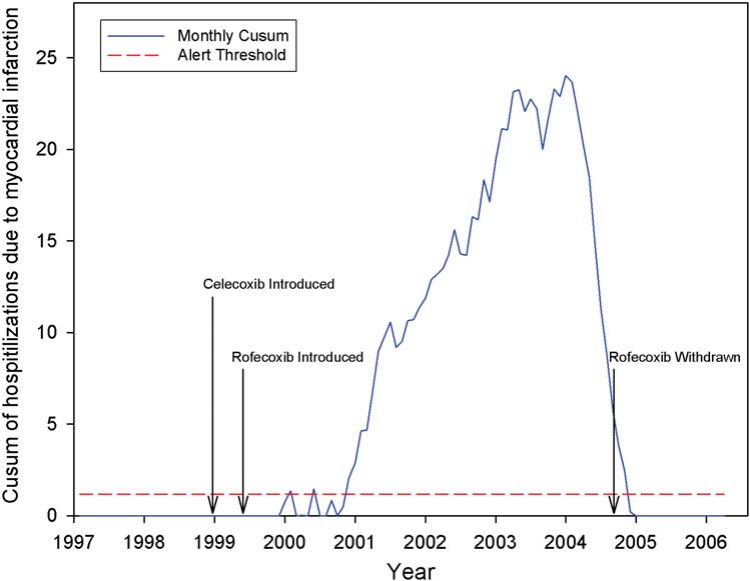
Cumulative sum (CUSUM) chart of monthly incidence of hospitalizations due to myocardial infarction from January 1, 1997 to March 30, 2006. Assigning 1997 and 1998 as a baseline period, a target mean of 47.1 hospitalizations due to myocardial infarctions per 100,000 and a standard deviation of 2.8 per 100,000.The threshold for CUSUM was calculated as 1.18, yielding an average-run-length of 50 (dashed red line). An aberration, two standard deviations from baseline, was initially detected in January of 2000, with a CUSUM value of 1.37 and 51.5 myocardial infarction-related hospitalization per 100,000 stays (solid line).

Based on the mean and standard deviation of MI incidence for a baseline period of 1997–1998, we first tested for cumulating deviations above a target mean of 47.1 MI-related hospitalizations per 100,000 and a standard deviation of 2.8 per 100,000. (Figure 1) The threshold for CUSUM was calculated as 1.18, yielding an average-run-length of 50. In other words, we set the false alarm rate to once per 50 months. An aberration of two standard deviations from baseline was initially detected in January of 2000, with a CUSUM value of 1.37 and 51.5 MI-related hospitalizations per 100,000 stays (9.3 percent above expected). This significant rise was eight months after the market introduction of rofecoxib and one year after the introduction of celecoxib. A second and sustained rise began November of 2000 with a CUSUM value of 2.08 and 54.2 MI-related hospitalizations per 100,000 (16 percent above expected). The exceedance peaked in March of 2003, 46 months after the introduction of rofecoxib with a CUSUM value of 16.5. Threshold was exceeded for 48 consecutive months, ending in October 2004, one month after the market withdrawal of rofecoxib.

Second, we modeled the impact of market availability of celecoxib and rofecoxib on inpatient stays for MIs. For celecoxib, inpatient stays due to MI were 41.6 per 100,000 prior to the December 1999 release (n = 24 months; 95 percent confidence interval, 40.4 to 42.8), and 47.4 from January 1999 to March 2006 (n = 87 months; 95 percent confidence interval. 46.3 to 48.6). MI-related hospitalizations were 41.7 per 100,000 over the period prior to rofecoxib introduction and post-withdrawal (n = 47 months; 95 percent confidence interval, 40.7 to 42.7). While rofecoxib was on the market, the rate of MI-related hospitalizations was 49.4 per 100,000 (n = 64 months; 95 percent confidence interval. 48.3 to 50.5), an 18.5 percent increase. We measured the effect of celecoxib and rofecoxib dispensing on the time series of MI-related hospitalizations by Poisson regression where the two drugs were included as indicator variables. The model for rofecoxib revealed a positive relationship with counts of MIs (rate ratio 1.14; 95 percent confidence interval, 1.09 to 1.19; P<0.001), explaining 28.7 percent of the deviance. The model for celecoxib also revealed a significant positive relationship with counts of MIs (rate ratio 1.09; 95 percent confidence interval, 1.01 to 1.18; P = 0.022), explaining 5.2 percent of the deviance.

**Figure 2:**
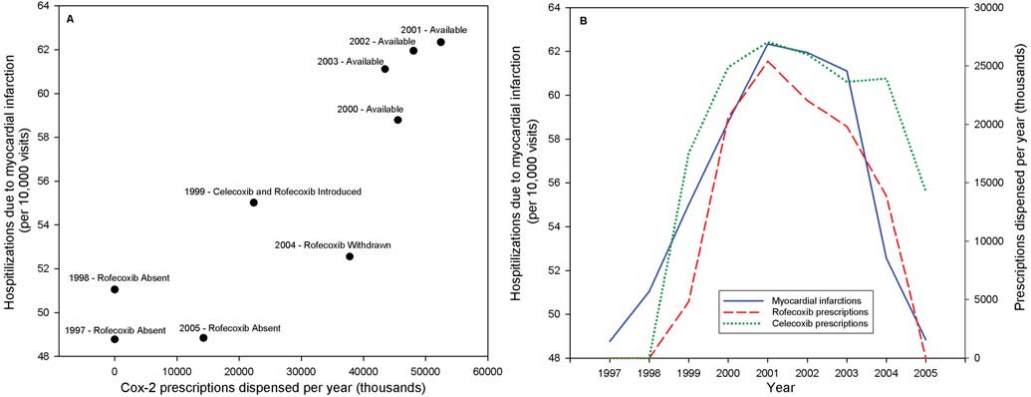
Association between yearly prescriptions for rofecoxib and celecoxib and incidence of myocardial infarctions. A) Scatter plot reveals the positive relationship between national prescriptions estimates for rofecoxib and celecoxib and hospitalizations due to myocardial infarction. Prescriptions for rofecoxib and celecoxib peaked in 2001 with 52,466,000. Incidence of stays due to myocardial infarction also peaked in 2001 with 62.3 hospitalizations per 10,000. B) Line graphs show the interrupted time series of myocardial infarction-related hospitalizations. Prescriptions for rofecoxib and celecoxib explained approximately 50.3 percent of the deviance in yearly variation of myocardial infarction related hospitalizations.

Finally, we measured the yearly impact of estimated celecoxib and rofecoxib use on hospitalizations due to MIs as a dose-response relationship using national prescription data (Table 1). Prescriptions for celecoxib and rofecoxib peaked in 2001 with 25.41 and 27.06 million prescriptions dispensed nationally, respectively. Incidence of MI-related hospitalizations also peaked in 2001 with 62.3 hospitalizations per 10,000 (Figure 2). Yearly counts of MIs were significantly predicted by prescriptions for rofecoxib (0.7% increase in MIs per million prescriptions; 95 percent confidence interval, 0.2% to 1.3%) and marginally for celecoxib (0.6% increase in MIs per million prescriptions, 95 percent confidence interval, −0.005% to 1.8%;). For every million prescriptions of both rofecoxib and celecoxib, we found a 0.5 percent increase in MI (95 percent confidence interval, 0.1 to 0.9), explaining approximately 50.3 percent of the deviance in yearly variation of MI-related hospitalizations.

**Table 1:** Yearly incidence in hospitalizations due to myocardial infarctions and corresponding national estimates of prescriptions for rofecoxib and celecoxib.

Year	Myocardial infarction-related hospitalizations*	Total inpatient†	Incidence of Myocardial infarction-related hospitalizations (per 10000)§	Rofecoxib scripts dispensed (millions)‡	Celecoxib scripts dispensed (millions)‡
1997	3475	89638	48.8	0	0
1998	3654	93562	51.0	0	0
1999	3958	99047	55.0	4.84	17.54
2000	4357	105426	58.8	20.63	24.91
2001	4644	105603	62.3	25.41	27.06
2002	4627	107898	61.9	22.04	26.01
2003	4569	110735	61.1	19.83	23.65
2004	3923	110677	52.6	13.87	23.92
2005	3641	111751	48.8	0	14.27

* Unique patients hospitalized for a myocardial infarction, having being assigned a primary or admitting diagnosis of myocardial infarction (International Classification of Diseases, Ninth Revision (ICD-9) code 410).

† Unique patients hospitalized at the Brigham and Women's Hospital and Massachusetts General Hospital

‡ Total numbers of prescriptions dispensed were obtained from the freely available IMS Health annual report on Commonly Requested Therapeutic Class and Product Information. Estimates are derived from a national prescription sales database.

§ Incidence is calculated using the estimated underlying population market share coverage by both study hospitals

**Figure 3:**
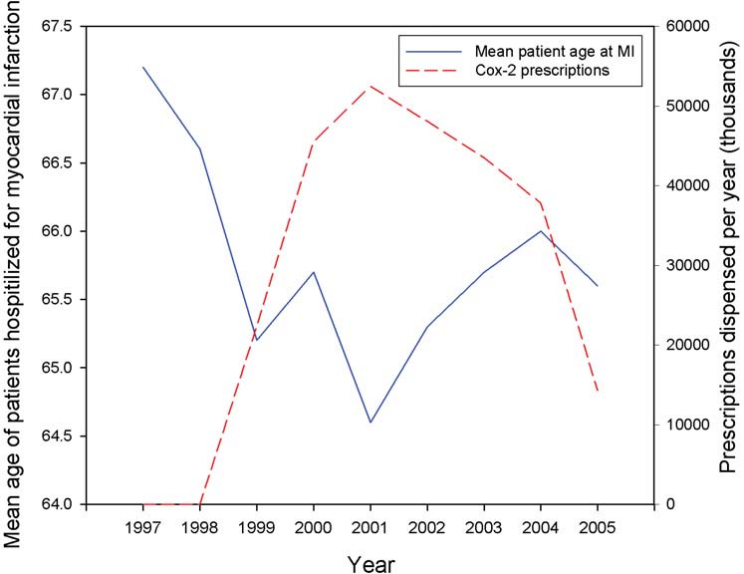
Association between yearly prescriptions for rofecoxib and celecoxib and mean age of patients hospitalized for myocardial infarctions. Line graph showing a significant negative association between mean patient age and national prescription volume for rofecoxib and celecoxib (Spearman correlation, 0.67, P<0.05). Prescriptions for rofecoxib and celecoxib peaked in 2001 with 27,060,000 prescriptions dispensed nationally while mean age was 64.6, the lowest in nine years.

We also find a significant negative association between mean patient age and prescriptions for celecoxib and rofecoxib (Spearman correlation, −0.67, P<0.05). Over the years when rofecoxib was not available, the mean age was 66.5 compared to a mean age of 65.4 over the period of market availability. Figure 3 shows the nadir in age corresponding to the peak of rofecoxib and celecoxib prescriptions.

### Anchor Diagnoses

To demonstrate that our findings were not simply based on changing healthcare utilization, we investigated temporal trends for hospitalizations for three other diagnoses. Though showing a positive linear increase, abdominal aortic aneurysm (Spearman correlation, 0.17; P = 0.66), appendicitis (Spearman correlation, 0.11; P = 0.78), and pneumonia and influenza (Spearman correlation, 0.071; P = 0.86) were not associated with trends in MI. Further, only trends in inpatient visits for MI were significantly correlated with national prescription rates for rofecoxib and celecoxib (Spearman correlation 0.93; P<0.001).

## Discussion

Our findings demonstrate striking longitudinal trends in the population rate of MI and strongly suggest a population-level impact of rofecoxib and celecoxib on MI. Temporal trends in inpatient stays due to MIs are tightly coupled to the rise and fall of prescription of COX-2 inhibitors, with an 18.5 percent increase in inpatient stays for MI during the time when rofecoxib was on the market. These results are consistent with prior reports demonstrating cardiovascular effects of rofecoxib and celecoxib. [9], [10], [11], [12], [13], [14], [15], [17], [27] Further, mean age at MI appears to have been lowered by use of these medications.

Using population surveillance methods [28], [29], [30] we confirm a sharp rise in adverse cardiovascular events within eight months of the market introduction of rofecoxib and one year after launching of celecoxib. A second rise in November 2000 occurred during the same month as the publication of the Vioxx Gastrointestinal Outcomes Research (VIGOR) study.[31], [32] The exceedence vanished within one month of the withdrawal of rofecoxib. The population-level findings, though based on aggregated data, are used to confirm a hypothesis based on clinical trial data [16] and to estimate the magnitude of the effect across the population.

We used two approaches to identify the impact of the prescriptions for COX-2 selective inhibitors. The first was to simply treat each drug as a binary (present or absent) variable. We find increases in inpatients stays for MI after the introduction of rofecoxib and celecoxib. Second, we applied a correlational approach to identify a dose-response relationship by modeling the temporal fluctuations in inpatient stay for MIs with national prescription rates. We find that adverse cardiovascular events coincide best with the time period of population exposure to rofecoxib and celecoxib. The analyses described are subject to the known pitfalls of ecological studies, with caveats about inferring causality.[33], [34] However, our results are especially convincing given the return to baseline following the market withdrawal of rofecoxib and our confirmatory analysis. Further, this ecological finding is confirming a prior suspected association.

Our study does have limitations. Our results, based only on an inpatient population, may underestimate population-level rates of MI. In our analysis, we treated individual patients as independent observations, narrowing confidence intervals for our rate ratio estimates. However, these error calculations do not account for the clustering of responses associated with temporal variation in risk. The IMS prescription data were based on national estimates and may not accurately represent possible local variation in Boston. For instance, geographic variation in prescription practices for rofecoxib and celecoxib may have been disproportionately affected by media attention regarding early reports of associated adverse cardiovascular events. However, our use of yearly estimates should smooth this potential bias. The prescription estimates also do not account for continued use of the drug after its withdrawal from the market.

While part of the power of this approach stems from availability of the full interrupted time series, including the period after market withdrawal of rofecoxib, our findings highlight the importance of understanding, modeling, and surveying for baseline adverse outcomes to detect population-level aberrations from that baseline. This study was performed with data from only two hospitals a in single health care system, in a single city. In an active post launch surveillance system, such signals will need to be rapidly evaluated and adjudicated with replication in other data sets, in other regions, and through multi-signal integration approaches.

High profile failures to detect safety problems during the pre-approval period have brought new intense scrutiny on the drug approval process.[35], [36], [37], [38], [39] Revelation of an association between rofecoxib and MI [32], [35], [40], [41] was a signal event highlighting catastrophic failures in monitoring the safety of therapeutics already on the market.[42] Because adverse effects of drugs and therapeutics are often not detected in Phase III clinical trials, [43] or they are suspected but not confirmed, a robust drug safety system requires ongoing monitoring of adverse events throughout the drug development lifecycle.[44] However, at present, safety efforts are not balanced across the lifecycle.

There is no generally accepted process or gold standard set of methods for identifying safety problems with drugs during the post launch phase. A major vehicle for hypothesis generation is the FDA's Adverse Event Reporting System (AERS) which collects voluntary reports[45] that are somewhat limited by poor quality, under-reporting, and skewed and inadequate ascertainment.[46], [47], [48], [49] Nonetheless, AERS-based findings are often the only basis for regulatory action.[49], [50], [51] Ultimately, however, it impossible to correct for inherent data limitations.

Notably, in the earlier part of the decade, it would have been very unlikely that a clinician would associate a MI with rofecoxib and hence to report it to the AERS system. Likewise, a clinician would have been unlikely to report a hip fracture in a patient taking a proton pump inhibitor.[52] There is an emerging consensus that semi-automated analysis of healthcare databases is the next logical step.[21], [22] Robust post-marketing surveillance requires a multimodality approach to monitoring the health of individuals using drugs and devices. As confirmatory evidence for the veracity of a suspected association, use of a population monitoring approach as an adjunct to pharmacovigilence methods might have helped provide earlier support for the market withdrawal of rofecoxib. Without a known prior association, population health monitoring could help generate hypotheses about candidate medication risk factors. This is especially important for widely prescribed medications where there is a substantial risk of population-level effects. If used prospectively, however, to look across drugs at patterns of prescribing and outcomes, careful attention would need to be paid to issues of specificity and multiple testing.
